# Taking care of inpatients with fragility hip fractures: the hip-padua osteosarcopenia (Hip-POS) fracture liaison service model

**DOI:** 10.1007/s40618-024-02425-z

**Published:** 2024-07-06

**Authors:** G. P. Arcidiacono, C. Ceolin, S. Sella, V. Camozzi, A. Bertocco, M. O. Torres, M. G. Rodà, M. Cannito, A. Berizzi, G. Romanato, A. Venturin, V. Cianci, A. Pizziol, E. Pala, M. Cerchiaro, S. Savino, M. Tessarin, P. Simioni, G. Sergi, P. Ruggieri, S. Giannini, the Hip-POS working group, G. P. Arcidiacono, C. Ceolin, S. Sella, V. Camozzi, A. Bertocco, M. O. Torres, M. G. Rodà, M. Cannito, A. Berizzi, G. Romanato, A. Venturin, V. Cianci, A. Pizziol, E. Pala, M. Cerchiaro, S. Savino, M. Tessarin, P. Simioni, G. Sergi, P. Ruggieri, S. Giannini, Carlotta Andaloro, Giulia Bano, Deris Gianni Boemo, Ester Bukli, Davide Cannavò, Alberta Cecchinato, Martina Dall’Agnol, Marina Rui, Mario Degan, Marta Dianin, Martin Diogo, Michela Ferrarese, Claudia Finamoni, Francesca Guidolin, Mario Rosario Lo Storto, Elena Marigo, Stefano Masiero, Caterina Mian, Maria Vittoria Nesoti, Mor Peleg Falb, Cristina Russo, Cristina Simonato, Giulia Termini, Hillary Veronese, Francesca Zanchetta, Chiara Ziliotto

**Affiliations:** 1 Clinica Medica 1, Department of Medicine, Azienda Ospedale-Università Padova, Padua, Italy; 2 Department of Medicine - DIMED, Division of Metabolic Disease (DIMED), University of Padova, Padua, Italy; 3 Geriatric Unit, Department of Medicine, University Hospital of Padova, Padua, Italy; 4 Department of Neurobiology, Care Sciences and Society, Aging Research Center, Karolinska Institutet and Stockholm University, Stockholm, Sweden; 5 Endocrinology Unit, Department of Medicine, Azienda Ospedale-Università Padova, Padua, Italy; 6 Orthopedics and Orthopedic Oncology Unit, Azienda Ospedale-Università Padova, Padua, Italy; 7 Orthopedics and Traumatology Unit, Azienda Ospedale-Università Padova, Padua, Italy; 8 Physical Medicine and Rehabilitation Unit, Azienda Ospedale-Università Padova, Padua, Italy; 9 Emergency Department, Azienda Ospedale-Università Padova, Padua, Italy; 10 Department of Medicine, Università Di Padova, Padua, Italy; 11 Department of Directional Hospital Management, Azienda Ospedale-Università Padova, Padua, Italy

**Keywords:** Fracture liaison service, Osteoporosis, Sarcopenia, Hip fractures, Fracture prevention

## Abstract

**Purpose:**

Osteoporotic fragility fractures (FF), particularly those affecting the hip, represent a major clinical and socio-economic concern. These fractures can lead to various adverse outcomes, which may be exacerbated by the presence of sarcopenia, especially among older and frail patients. Early identification of patients with FF is crucial for implementing effective diagnostic and therapeutic strategies to prevent subsequent fractures and their associated consequences.

**Methods:**

The Hip-POS program, implemented at Azienda Ospedale-Università Padova, is a Fracture Liaison Service (FLS) program to evaluate patients aged > 50 years old admitted with fragility hip fractures, involving an interdisciplinary team. After the identification of patients with hip fractures in the Emergency Department, a comprehensive evaluation is conducted to identify risk factors for further fractures, and to assess the main domains of multidimensional geriatric assessment, including muscle status. Patients are then prescribed with anti-fracture therapy, finally undergoing periodic follow-up visits.

**Results:**

During the first five months, a total of 250 patients were evaluated (70.4% women, median age 85 years). Following assessment by the Hip-POS team, compared to pre-hospitalization, the proportion of patients not receiving antifracture therapy decreased significantly from 60 to 21%. The prescription rates of vitamin D and calcium increased markedly from 29.6% to 81%.

**Conclusions:**

We introduced the Hip-POS program for the care of older adults with hip fractures. We aspire that our model will represent a promising approach to enhancing post-fracture care by addressing the multifactorial nature of osteoporosis and its consequences, bridging the gap in secondary fracture prevention, and improving patient outcomes.

## Introduction

Osteoporosis is a chronic condition characterized by reduced bone mass and deterioration of the bone microarchitecture, resulting in an increased risk of fragility fractures (FF) [1]. These are defined as bone damage resulting from low-energy trauma, i.e. a force equivalent to a fall from standing height or less [2]. With the progressive aging of the population, Italy expects to see a 23% increase in FF incidence between 2017 and 2030, in line with the estimated trend in other European countries over the same period [3]. In particular, the number of hip fractures among Italian women is expected to increase from 82,060 to 98,539, with a corresponding rise in economic costs of 27% [3].

One of the aspects of osteoporosis of greatest concern is the impact that FF have on disability and mortality. Following a hip fracture, 30% of patients are left with a permanent disability, while mortality rates vary from 15 to 25% [4]. Therefore, early identification of patients who have had a FF is crucial to developing an effective diagnostic and therapeutic strategy to prevent subsequent fractures and their consequences. This has even greater importance in light of the fact that fractures themselves represent a considerable risk factor for refracture [5], the incidence of which is exceptionally high within one year of a first fracture at any site [6]. Moreover, despite the availability of various treatments with proven robust efficacy [7–9], an insufficient percentage of patients are assigned to secondary therapy for the prevention of osteoporosis, creating a notable "secondary fracture prevention gap" [10], and contributing to a significant increase in morbidity, mortality, and healthcare economic costs [11].

For these reasons, several post-fracture care (PFC) programs have been developed over the years. They include, for example, orthogeriatric models and fracture liaison services (FLS) [10]. FLS programs deal with secondary prevention of fractures, focusing on both out-patients and hospitalized patients with a prevalent fracture. A range of professional figures may be involved in FLS, such as orthopedic surgeons, internists, endocrinologists, rheumatologists, geriatricians, physiatrists, radiologists, and pain physicians [12]. Although the effectiveness of these models has been widely demonstrated [13], they still present some challenges. One of these is the assessment of muscle mass and quality, which is crucial for correctly estimating the cumulative risk of further fractures. The negative consequences of FF may be exacerbated in the presence of muscle deficits, which not only influence the risk of falling, but also slow down motor recovery after a fracture [14]. In recent decades greater attention has therefore been paid to the musculoskeletal unit rather than just the bone, and the term "osteosarcopenia" has been coined [15]. Sarcopenia, together with osteoporosis, negatively affects balance, and both the risk and fear of falling [14]. Furthermore, it is a well-known risk factor for prolonged hospitalization, infection, frailty, disability, and death [14].

Given these premises, in this paper we present our FLS model, Hip-POS. Its aim is the comprehensive and interdisciplinary care of patients with fragility hip fractures during hospitalization and throughout their subsequent outpatient follow-up, focusing specifically on the osteo-muscular unit.

## Methods

### Hip-POS FLS Program design and purpose

Our Hip-POS program was established at the Azienda Ospedale-Università Padova (Italy) bringing together various healthcare professionals involved in the care of patients with fragility hip fractures. The Hip-POS model is a diagnostic and therapeutic pathway consisting of various phases, from referral of the fractured patient on first admission to the Emergency Department, to managing care during the hospital stay, multidimensional assessment, prescription of appropriate personalized therapy, and outpatient follow-up. The inclusion criteria for enrollment in our Hip-POS program are: 1) age 50 years or older, and 2) hospital admission for a fragility hip fracture. The exclusion criterion is the presence of traumatic or pathological fractures.

The interdisciplinary team includes the following figures, each with specific roles:

- Endocrinologists and internists for diagnostic assessment of skeletal fragility, identification of potential secondary causes of osteoporosis, and prescription of an appropriate pharmacological anti-fracture treatment.
- Geriatricians for multidimensional patient evaluation, with a specific focus on comorbidities, functional autonomy, and nutritional and muscular status.
- Orthopedic surgeons for the management of surgical treatment and wound healing.
- Physiatrists to design rehabilitation programs to ensure rapid functional recovery of the patient.
- Qualified and adequately trained nursing personnel to support medical management of the patient, for example, by identifying candidates for the FLS project, overseeing the organization of assessments and examinations, and coordinating data management.

The Hip-POS model has the following aims:

- To identify and promptly address conventional risk factors for skeletal fragility, and especially additional risk factors for frailty, through the analysis of the main domains of the multidimensional geriatric assessment (i.e., cognitive, social, functional, and nutritional statuses, as well as polypharmacy).
- To validate an objective tool for evaluating patients who could most benefit from osteoporosis therapy, based on an estimated one-year prognosis.
- To guarantee a sufficient degree of functional autonomy by identifying states of sarcopenia and encouraging rapid mobilization with the participation of physiatrists.

Specifically, these objectives will be pursued by investigating the following outcomes at 6 and 12 months after discharge:

- Functional autonomy, rehospitalizations, and mortality.
- Patient adherence to anti-fracture therapy.
- Recurrence of new fragility fractures.

Approval for the project was obtained from the local Ethical Committee (Comitato Etico Territoriale Area Centro-Est Veneto, 442n/AO/23) in accordance with the principles outlined in the Declaration of Helsinki in order to ensure compliance with ethical standards in research involving human subjects. Upon hospital admission, consent is obtained from the patients to collect their data for the purposes of the FLS project. Patients are informed that declining consent will not affect their access to current standards of diagnostic and therapeutic care. All patient data are stored on a dedicated computer platform in anonymized form.

### Data collection

The Hip-POS model involves carrying out the following assessments and tests:

- *Patient assessment*: Physiological, clinical and pharmacological data are collected during a medical interview by skilled physicians. Comorbidities are assessed with the Cumulative Illness Rating Scale (CIRS) [16]. The patient’s functional autonomy is assessed with the Activities of Daily Living (ADL) [17] and the Instrumental Activities of Daily Living (IADL) [18], nutritional status with the Mini Nutritional Assessment (MNA) [19], and cognitive performance with the Short Portable Mental Status Questionnaire (SPMSQ) [20]. The Exton Smith scale (ESS) is used to determine the risk of developing pressure sores [21]. Finally, the Multidimensional Prognostic Index (MPI) is calculated from information obtained from the SPMSQ, ESS, ADL, IADL, MNA, CIRS, the number of drugs taken by the patient, and cohabitation status in order to predict mortality [22]. An integrated assessment of risk factors for skeletal fragility is performed by calculating the ten-year probability of major osteoporotic fractures through a validated calculator, such as FRAX (Fracture Risk Assessment Tool), the most widely-used algorithm, or the Italian FRAX-derived tool, DeFRA [23].
- *Anthropometry and muscle strength measurement*. The following measures are recorded within 24–48 h of patient admission: body weight and height [24], body mass index (BMI), calf circumference [25], and mid-upper arm circumference [26]. Upper limb strength is evaluated with electronic hand dynamometers by trained medical personnel, the final measure being the mean maximum performance by the dominant and the non-dominant hands.
- *Laboratory investigations*. First and second tier laboratory investigations recommended by the Italian Guidelines for the diagnosis, prevention and management of osteoporosis [9] are carried out on evaluated patients.
- *Evaluation of bone mineral density (BMD) and vertebral fractures*. BMD is assessed by dual-energy X-ray absorptiometry (DXA) at the lumbar spine and at the contralateral proximal hip to the one affected by the fracture (femoral neck and total hip). The presence of vertebral fractures in the patient is determined from radiographs of the dorsal and lumbosacral spine in lateral projection. Vertebral fractures are classified with the Genant semi-quantitative method [27].
- *Evaluation of body composition*. Body composition is assessed by whole-body tetrapolar bioelectrical impedance analysis (BIA). Estimates of fat-free mass (FFM) and appendicular skeletal muscle mass (ASMM) are calculated using the equations developed by Kyle et al. [28]. The skeletal appendicular muscle mass index (ASMMI) and the fat mass index (FMI) are obtained by dividing the ASMM and FM, respectively, by the subject’s height in meters squared. Patients with fever, severe dehydration, heart failure accompanied by significant body edema, or with unstable medical conditions (such as uncontrolled cardiac arrhythmias, uncontrolled hypertension, recent myocardial infarction or angina pectoris, hemodynamic instabilities, or severe dementia) that may alter the results of the bioimpedance examination are excluded.

### Phases

**Fig. 1:**
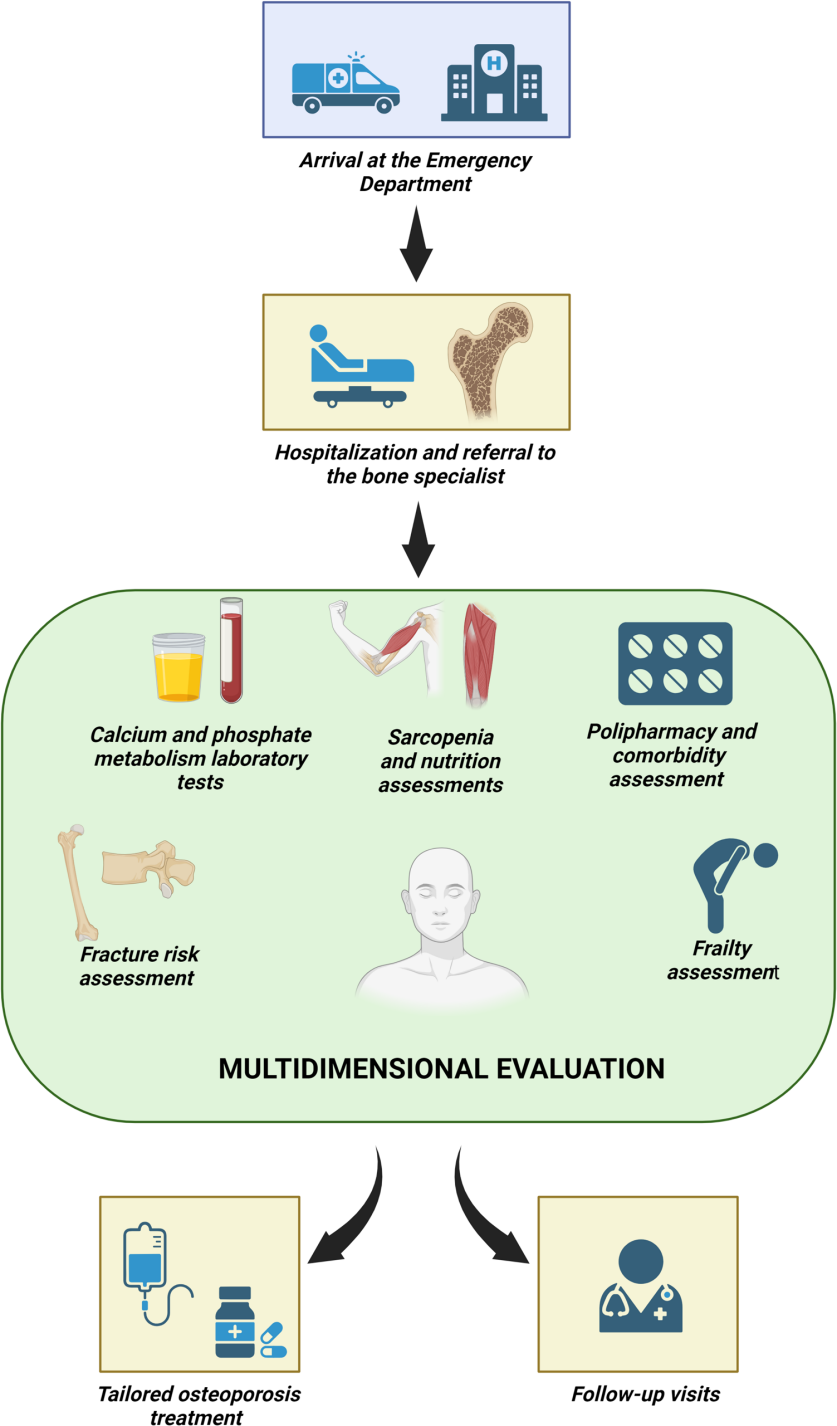
Description of the Hip-POS model

The Hip-POS model consists of the following phases (Fig. 1 and Table 1):

1. Patient identification: The patient identification process begins in the Emergency Department of the Azienda Ospedale-Università Padova. Patients presenting with suspected hip fractures undergo comprehensive clinical and radiological assessments to confirm or exclude the fracture. When hip fracture is confirmed, the patient is admitted to the Orthopedics Department, where surgical intervention is conducted in accordance with recommended timelines [29]. Patients who meet the enrollment criteria are automatically referred electronically to the program coordinator upon admission and are offered the opportunity to participate in the Hip-POS program, for which informed consent is to be obtained. All program participants, regardless of their level of frailty, are assessed by a bone specialist during their hospitalization.
2. Fracture risk stratification: Before prescribing patients a specific osteoporosis therapy they should be assessed for the presence of low BMD and/or concurrent vertebral fractures. If it is not feasible to conduct these assessments during hospitalization, it is strongly recommended that they be carried out in the subsequent three months, and that the results be available for review during outpatient check-ups. In addition, potential risk factors for osteoporosis are thoroughly assessed using FRAX or DeFRA. Finally, first-level blood and urine investigations are conducted to assess the skeletal metabolism of all patients. Based on the individual patient’s clinical suspicion and MPI score, and, consequently, the one-year prognosis, second-level laboratory investigations may also be carried out to rule out any further potential secondary causes of skeletal fragility.
3. Assessment of patients’ frailty status and prognosis using a multidimensional tool: The patient’s clinical complexity is evaluated by the MPI score, one of the most widely used prognostic tools for hospitalized older adults. The MPI covers various dimensions of frailty, including nutritional and cognitive status, polypharmacy, cohabitation status, and comorbidities. The accuracy of the MPI establishes it as a dependable tool for stratifying a population based on short- and long-term mortality risk: If the index falls within the range of 0 to 0.33, the prognostic risk of mortality at 12 months is deemed low; if it falls between 0.34 and 0.66, the prognostic risk is considered moderate; between 0.67 and 1.00, the prognostic risk is severe. Given the high prevalence of sarcopenia among geriatric patients, strength and muscle mass are also evaluated. Patients identified as malnourished or sarcopenic are given a suitable dietary regimen in accordance with The European Society for Clinical Nutrition and Metabolism (ESPEN) guidelines [30] to ensure the correct intake of protein and calories. Finally, patients and their caregivers are given instructions and recommendations for fall prevention during hospitalization or upon discharge. The efficacy of these recommendations is assessed during outpatient follow-ups, where the same tests (bioimpedance examination and dynamometer tests) are repeated to evaluate any improvements.
4. Intervention*:* Following the comprehensive, interdisciplinary assessment outlined in the previous phases, all patients are considered for calcium and vitamin D supplementation based on their estimated daily intake and measured serum levels. Recommendations for anti-fracture therapy are in line with national [9] and international guidelines [7, 8], as well as with Italian prescribing regulations [31]. The choice of anti-fracture therapy depends on the underlying cause of the skeletal fragility, comorbidities, the extent of the patient’s autonomy, and the presence of any contraindications to specific drug classes.
5. Outpatient follow-up: All patients who consent to enrolment in the FLS program undergo periodic follow-ups, either through telephone calls or in-person visits according to the patient’s condition. In the first year, follow-ups will be conducted at 6 and 12 months after the index fracture. After the first year, follow-ups will be individually tailored to each patient, taking into account their level of comorbidity, fracture risk, and potential therapeutic alternatives. During the follow-up appointments, the patient will be assessed for adherence to the prescribed therapy, the presence of any adverse effects, the occurrence of subsequent fractures following the index fracture, the necessity for further hospital admissions, the level of residual functional autonomy, the risk of falls, and the potential need for a caregiver or admission to an elderly care facility.

**Table 1:** Macrophases of the Hip-POS model and the various specialized skills involved

WHEN	PHASE	Description	Specialists in charge
ON ADMISSION	Emergency department	• Suspected hip fracture: comprehensive clinical and radiological assessments to confirm or exclude the fracture • Hip-POS inclusion criteria: age ≥ 50 years; no traumatic or pathological fractures • Patients meeting the inclusion criteria are electronically referred to the program coordination team upon admission	Emergency department physicians
	Fracture risk stratification	• Fracture risk stratification: FRAX and/or DeFRA • Medical history and laboratory evaluation are carried out to exclude secondary causes of osteoporosis • Dorso-lumbar spine X-ray radiographs are taken to identify possible vertebral fractures	Geriatrician, internist, endocrinologist
	Multidimensional evaluation	• The patient’s clinical complexity is assessed using the MPI score, which is the sum of ADL, IADL, SPMSQ, CIRS, ESS, MNA, total drugs, and cohabitation status	Geriatrician, internist, endocrinologist
	Sarcopenia assessment	• Whole-body tetrapolar BIA is used to estimate FFM and ASMM and their respective indices	Geriatrician, physiatrists
DURING HOSPITAL STAY	Osteoporosis therapy	• Therapy is prescribed according to current guidelines, taking into account the patient's prognosis, i.e. MPI scores • All patients whose blood and urine tests show calcium and vitamin D deficiencies are advised to take supplements	Geriatrician, internist, endocrinologist
AT DISCHARGE	Dietary recommendation and fall prevention	• Dietary recommendations are given following ESPEN guidelines • Patients and caregivers are given fall prevention education	Geriatrician
OUTPATIENT FOLLOW-UP	Sarcopenia assessment	• Whole-body tetrapolar BIA is used to estimate FFM and ASMM and their respective indices	Geriatrician, physiatrists
	Osteoporosis visit	• Calcium and phosphate metabolism laboratory tests, a new dorsal-lumbar spine X-ray, and bone densitometry • Therapy is adjusted according to the patient's risk of fracture, needs, and comorbidities	Geriatrician, internist, endocrinologist

*FRAX* fracture risk assessment tool, *DeFRA* derived FRAX, *MPI* multidimensional Prognostic Index, *BIA* bioelectrical impedance analysis, *ADL* activities of daily living, *IADL* instrumental ADL, *SPMSQ* Short Portable Mental Status Questionnaire, CIRS Cumulative illness rating scale, *ESS* exton smith scale, *MNA* mini nutritional assessment, *FFM* fat-free mass, *ASMM* appendicular skeletal muscle mass, *ESPEN* the European society for clinical nutrition and metabolism

## Results

We present the preliminary data from the first five months of Hip-POS’s activity. Table 2 summarizes the key characteristics of patients evaluated by our team, categorized by sex. We assessed 250 patients, of whom 70.4% were women, with an average age of 85 years. Men were more frequently active smokers (14.9% vs. 6.3%, p = 0.03) and were more commonly affected by diabetes, Chronic Obstructive Pulmonary Disease (COPD), and cirrhosis. Conversely, women had a higher incidence of previous clinical vertebral fractures, although this difference was not statistically significant. The mean MPI value was 0.41 ± 0.23, with no significant differences between males and females (p = 0.10). There was a notable increase in treated patients, rising from approximately 40% to around 80% after evaluation by the Hip-POS team. Specifically, only 26 patients (23 women and 3 men) received treatment before their index hip fractures, including oral and IV bisphosphonates (73.1% and 19.2%, respectively) and denosumab (7.7%). During hospitalization, the Hip-POS team most frequently prescribed antiresorptive therapy (bisphosphonates: 87.2%; denosumab: 7.7%). Anabolic treatments had an overall prescription rate of 5.1%. In addition to fracture treatment, following our evaluations, approximately 81% of patients received vitamin D and calcium supplementation, compared to just 29.6% who had received such supplementation prior to admission.

**Table 2:** Characteristics of the sample

	Male patients (N = 74)	Female patients (N = 176)	p-value
Age (years)	84.5 (77.0–89.1)	85.0 (79.0–90.0)	0.54
Risk factors for skeletal fragility			
Previous hip fractures, n (%)	4 (5.4)	12 (6.8)	0.68
Previous clinical vertebral fractures, n (%)	8 (10.8)	35 (19.9)	0.08
Previous other fragility fractures, n (%)	14 (18.9)	47 (26.7)	0.19
Family history of osteoporosis, n (%)	15 (20.3)	28 (15.9)	0.40
Current smoking, n (%)	11 (14.9)	11 (6.3)	**0.03**
Glucocorticoid use, n (%)	2 (2.7)	6 (3.4)	0.77
Comorbidities			
Arterial hypertension, n (%)	53 (71.6)	120 (68.2)	0.59
Chronic heart failure, n (%)	13 (17.6)	32 (18.2)	0.91
Diabetes mellitus, n (%)	20 (27.0)	27 (15.3)	**0.03**
COPD, n (%)	11 (14.9)	10 (5.7)	**0.02**
Rheumatic Diseases, n (%)	2 (2.7)	10 (5.7)	0.31
CKD stages IV-V, n (%)	5 (6.8)	6 (3.4)	0.24
Esophagitis and/or peptic ulcer disease, n (%)	9 (12.2)	22 (12.5)	0.94
Liver cirrhosis, n (%)	7 (9.5)	5 (2.8)	**0.03**
Treatment before index hip fracture			**< 0.01**
No treatment, n (%)	57 (77.0)	93 (52.8)	
Only vitamin D supplements, n (%)	14 (18.9)	60 (34.1)	
Antifracture treatment with or without vitamin D supplements, n (%)	3 (4.1)	23 (13.1)	
Treatment prescribed after FLS evaluation			
Antifracture treatment with or without vitamin D supplements, n (%)	58 (78.4)	140 (79.5)	0.84

*CKD* chronic kidney disease, *COPD* chronic obstructive pulmonary disease, *FLS *fracture liaison service. Numbers are shown as median (interquartile range) or numbers (percentages), as appropriate

## Discussion

Fragility fractures, especially fragility hip fractures, are of substantial clinical and socio-economic concern, and have negative consequences for both mortality rates and functional autonomy among affected patients [32]. Without appropriate pharmacological anti-fracture treatment, an incident hip fracture is predictive of a second hip fracture, which occurs in up to 33% of patients and within approximately 1.5 years in half of these cases [6]. FLS models have proven to be effective in increasing the prescription of anti-fracture treatments and adherence to them, thereby reducing the risk of refracture and mortality rates [13].

In this paper, we have presented a novel FLS model that we established in Padua, which focuses on the individual patient—very often old and frail—along a pathway that encompasses referral, in-hospital clinical, laboratory and multidimensional assessments, initiation or prescription of anti-fracture therapy, and subsequent periodic follow-up. Among the advantages of our model is the electronically-based automatic referral of all patients admitted with a hip fracture to the program coordination section, where their skeletal fragility can be assessed within 24–48 h of hospital admission. This helps avoid the risk of diagnostic and therapeutic exclusion of the frailest patients, who might not attend subsequent outpatient appointments.

In our FLS model, we opted to use the MPI score to evaluate the degree of patient frailty. This is a multidimensional index derived from a comprehensive geriatric assessment and is currently validated as a prognostic tool for older populations in decision-making processes related to common interventions (e.g. transcatheter aortic valve implantation – TAVI) and in the management of specific acute and chronic conditions [33]. The MPI is considered a surrogate index of frailty in older, especially hospitalized, patients with various negative outcomes, including disability, a higher risk of re-hospitalization, and mortality [34]. Although there are other indices predicting 30 day and 1 year prognoses, such as the Nottingham Hip Fracture Score [35], by adopting the MPI in our FLS model we can assess multiple additional domains. Multidimensional assessment has been previously integrated into orthopedic management of old patients in orthogeriatric models, and can be useful in identifying frail individuals at higher risk of fractures [34]. In apparent contrast to this, our preliminary results identified a majority of patients with low MPI values, indicating lower overall frailty and, notably, a favorable one-year prognosis. The planned follow-ups will be useful for investigating the functional outcomes of these patients and determining any clinical developments in relation to the degree of frailty recorded during hospitalization.

Alongside multidimensional clinical assessment, first-level laboratory tests for bone fragility are carried out on all patients during the first days of hospitalization. On the basis of the individual patient’s clinical suspicion, second-level laboratory investigations may also be performed to rule out any further potential secondary causes of skeletal fragility. This may contribute to suspicion or diagnosis of the presence of bone fragility conditions other than osteoporosis, such as osteomalacia, hyperparathyroidism, or plasma cell disorders, which are common in older subjects [36]. Based on the patient’s estimated dietary intake and laboratory tests, vitamin D and calcium supplements may also be prescribed or administered during hospitalization, as they have been shown to correlate with a lower risk of refracture and mortality when combined with anti-fracture therapy [37].

Consistent with our preliminary findings, globally implemented FLS models have shown promising results in prescribing anti-fracture therapy. A meta-analysis highlighted substantial improvements in the prescription rates of osteoporosis medications within FLS groups compared to control groups. This trend is particularly notable in countries like Sweden, Italy, and the United Kingdom, where these models are managed by medical and nursing professionals [38]. The choice of pharmacological anti-fracture therapy depends on the underlying cause of the skeletal fragility, and the patient’s clinical status, frailty, and future fracture risk, as defined by the main national [9] and international guidelines [7, 8], and in accordance with the Italian health system’s prescribing regulations [31]. For patients at risk of low therapeutic adherence at home, preference is given to the administration of parenteral anti-fracture therapy, such as one-shot i.v. bisphosphonates, during the hospitalization period, unless contraindicated, to mitigate the potential risk of therapeutic exclusion. The use of zoledronic acid in older patients with a recent hip fracture has long been subject to debate because it was believed to alter the fracture healing process. However, in recent years several studies have concluded that bisphosphonate infusion does not in any way alter the healing process [39–43]. Moreover, the timing of zoledronic acid treatment has no effect on the incidence of complications in older patients with recent femoral fractures [40], so early administration during hospitalization under the supervision of a multidisciplinary team should, in our opinion, be encouraged.

Besides these factors, our FLS model aligns with the objectives of current existing models, i.e. an improvement in long-term adherence to osteoporosis treatments [44], a reduction in subsequent fractures, and improved mortality outcomes [13], as well as favorable cost-effectiveness [45]. As demonstrated by our preliminary results, the Hip-POS model was effective in increasing the anti-fracture treatment rate compared to that before the index fracture. We expect that our model, compared with others described in the literature, including those developed in Italy [12], will be better able to assess any additional benefits that may arise from multidisciplinarity and the additional focus on muscular health for medium- and long-term outcomes, which will need to be evaluated over time.

## Conclusions

The increasing prevalence of osteoporosis, attributable to a progressively ageing population, is a clinical and socio-economic problem that requires integrated and innovative solutions to reduce the negative outcomes associated with FF, particularly in older and frail patients. Our model incorporates a multidisciplinary approach to caring for patients with hip FF, which ensures they receive a personalized strategy that begins with the initiation of anti-fracture therapy and continues with ongoing patient monitoring. It also addresses often overlooked factors, such as nutrition, muscle status, and functional recovery, which play a crucial role in the prevention of secondary hip fractures. The potential long-term benefits of our model need to be evaluated over time, taking into account its impact on patient outcomes, economic viability, and potential integration with local health networks and primary healthcare services.

## Data Availability

All data generated or analyzed during this study are included in this published article.
